# Parathyroid adenoma in pregnancy: A case report and systematic review of the literature

**DOI:** 10.3389/fendo.2022.975954

**Published:** 2022-10-17

**Authors:** I. Pliakos, A. Chorti, Moysis Moysidis, G. Kotsovolis, T. Kaltsas, A. Pana, A. Ioannidis, TS. Papavramidis

**Affiliations:** ^1^ Department of Minimal Invasive Endocrine Surgery, Kyanous Stavros, Euromedica Clinic, Thessaloniki, Greece; ^2^ 1st Propedeutic Department of Surgery, AHEPA University Hospital, Faculty of Health Science, Medical School, Aristotle University of Thessaloniki, Thessaloniki, Greece; ^3^ 1st Department of Obstetrics and Gynecology, Papageorgiou Hospital, Faculty of Health Science, Medical School, Aristotle University of Thessaloniki, Thessaloniki, Greece

**Keywords:** parathyroid adenoma, pregnancy, endocrine surgery, parathyroid surgery, primary hyperparathyroidism

## Abstract

**Objective:**

Primary hyperparathyroidism is a common disorder of the parathyroid glands. Parathyroid adenoma (PA) in pregnancy is a relatively rare disease, whose diagnosis and treatment is a challenging task. The aim of the present study is to present a new case of parathyroid adenoma during pregnancy and to give a detailed account of all reported cases of parathyroid adenoma during pregnancy in the literature.

**Study design:**

A bibliographic research was performed, and characteristics of parathyroid adenomas in pregnancy such as age, gestational week at diagnosis, ionized calcium levels, genetic testing result, symptomatology, radiological method of localization, treatment method, gestational week at operation, and maternal/fetal complications were recorded.

**Results:**

A 34-year-old woman at her 25 weeks’ gestation was diagnosed with parathyroid adenoma and was referred to our Surgical Department due to contraindication for conservative treatment. A parathyroidectomy was performed, and the maternal and fetal postoperative period was uneventful. Two hundred eleven cases of parathyroid adenoma in pregnancy were recorded in the literature, and statistical analysis was performed. The median gestational week at diagnosis was 21 ± 9.61 weeks. The mean level of ionized calcium was 2.69 mmol/l [SD = 0.75 (2.55–2.84 95% CI)]. Most cases were familiar (72.4%), while surgery was the preferred treatment option (67.3%). The majority of cases were asymptomatic (21.7%), and the main radiological method applied for localization was ultrasound (63.4%).

**Conclusion:**

Parathyroid adenoma in pregnancy is a rare condition. The early diagnosis is of great importance as surgical treatment at the second trimester of pregnancy outweighs the maternal and fetal risks.

## Introduction

Primary hyperparathyroidism (PHPT) is a relatively common disorder of the parathyroid glands, causing skeletal, renal, and cardiac complications. As etiological features of PHPT, single adenoma, hyperplasia, carcinoma, and familiar causes (MEN—multiple endocrine neoplasia, FHH—familiar hypocalciuric hypercalcemia, hyperparathyroidism—jaw tumor syndrome) are presented (1). Primary hyperparathyroidism in pregnancy has an incidence of 1%, which is underestimated due to the plethora of undiagnosed cases (2, 3). The main symptomatology may mimic this of pregnancy, although severe maternal and fetal complications occur in untreated hypercalcemia. Mild hyperparathyroidism could be treated conservatively, but in cases of severe or symptomatic hypercalcemia, parathyroidectomy is the treatment of choice.

The aim of the present study is to present a new case of parathyroid adenoma during pregnancy and its diagnostic and therapeutic management. Furthermore, we aimed to give a detailed account of all reported cases of PA during pregnancy in the literature and to analyze the available data.

## Case report

**Figure 1:**
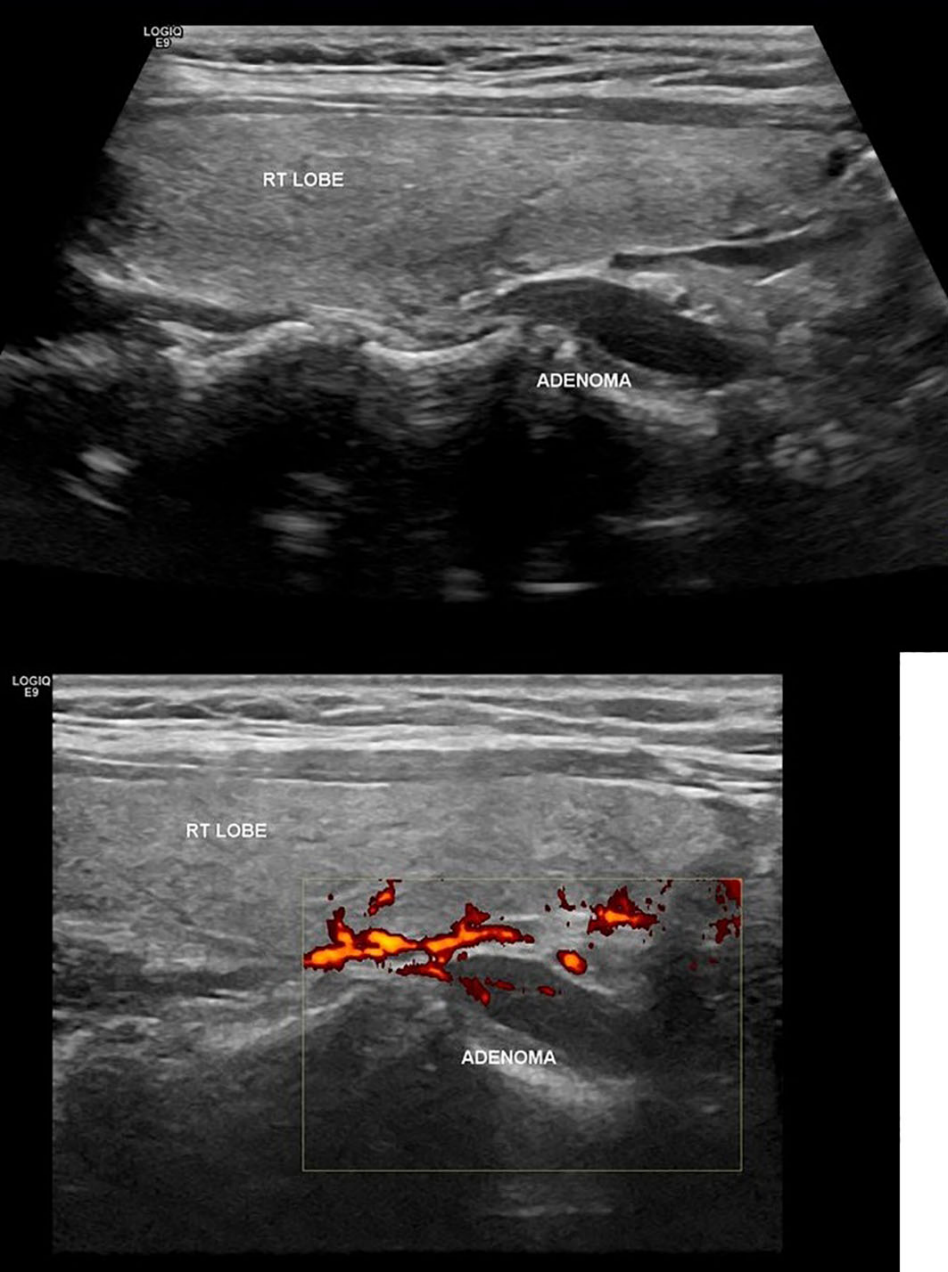
Ultrasonographic findings of parathyroid adenoma.

A 34-year-old woman at her 25 weeks’ gestation was referred to our Department of Endocrine Surgery due to a parathyroid adenoma diagnosed by her endocrinologist. The main symptom was hyperemesis during her second trimester of pregnancy. It is essential that she was free of symptomatology during her first trimester of pregnancy, and hyperemesis of the second trimester troubled her gynecologist, who referred her to the endocrinologist. Her past medical history was unremarkable, and her family history revealed no evidence of familiar endocrine disease. Her initial laboratory examination revealed a high ionized serum calcium level of 1.47 mmol/l (normal range 1.15–1.33 mmol/l), an increased parathyroid hormone (PTH) level of 75.8 pg/ml (normal range 15–65 pg/ml), a decreased serum phosphorus level of 2.4 mg/dl (normal range 2.7–4.5 mg/dl), an increased 24-h urine calcium level of 592 mg/24 h (normal range 100–300 mg/24 h), and a decreased vitamin D level 6.15 ng/dl (normal range >20 ng/dl). She was not advised for oral supplementation with vitamin D due to hypercalciuria and the risk of renal complications. Cervical ultrasonography revealed a flattened, hypoechoic, solid, homogenous nodule, located under the inferior pole of the right lobe of the thyroid gland, on its posterior part and proximal to the trachea, measuring 25 × 6.3 × 10 mm, indicative of parathyroid adenoma (**Figure 1**). The patient was advised about surgical treatment, as conservative treatment with vitamin D supplements was not an option and the parathyroid adenoma increased the risk for both preeclampsia and abortion. Her preoperative laboratory results revealed a total serum calcium level of 10.7 mg/dl (normal range 8.5–10.2 mg/dl), a serum phosphorus level of 2.6 mg/dl (normal range 2.6–4.5 mg/dl), and a PTH level of 78.1 (normal range 15–65 mg/dl). She underwent parathyroidectomy, during which the right superior parathyroid gland was found enlarged and was resected. The intraoperative PTH levels at the beginning of the operation and 15 min after the specimen removal were 176.9 and 30.29 pg/dl, respectively. The postoperative maternal and natal courses were uneventful. Chvostek and Trousseau signs were negative. Postoperative laboratory examination 8 h after the operation showed a serum calcium level of 9.4 mg/dl, a PTH level of 5.59 pg/dl, and a phosphorus level of 3.3 mg/dl. The patient was treated with intravenous calcium supplementation with 6 amp of calcium gluconate (1 amp = 1 g calcium gluconate) in 500 cc of 0.9% normal saline and oral calcium and vitamin D3 supplements (continued upon discharge). The following day, her laboratory exams were improved (Ca: 9.0 mg/dl, P: 3.1 mg/dl, PTH: 2.06 pg/dl), and after the advisory of her endocrinologist and gynecologist, N/S 1000 ml with 10 amp calcium gluconate was administered to the patient in order to avoid hypocalcemia and the possible maternal and fetal complications. The second postoperative day, her laboratory findings were Ca: 9.4 mg/dl, P: 4.7 mg/dl, and PTH: 3.71 pg/dl and she was discharged. The histological report concluded to a parathyroid adenoma measuring 2.4 cm and weighting 2.5 g without evidence of malignancy. A genetic testing was done postoperatively. The decision to proceed to surgery without genetic testing results was made as the patient was diagnosed in the second trimester, and the case demanded an urgent therapy by operation in the same trimester. The genetic testing results would be delayed 3 months, so we proceeded to surgery and the genetic results afterward were negative for MEN syndrome. During the postoperative follow-up period, she had an uneventful vaginal delivery and both mother and the newborn were normocalcemic.

## Methods

**Figure 2:**
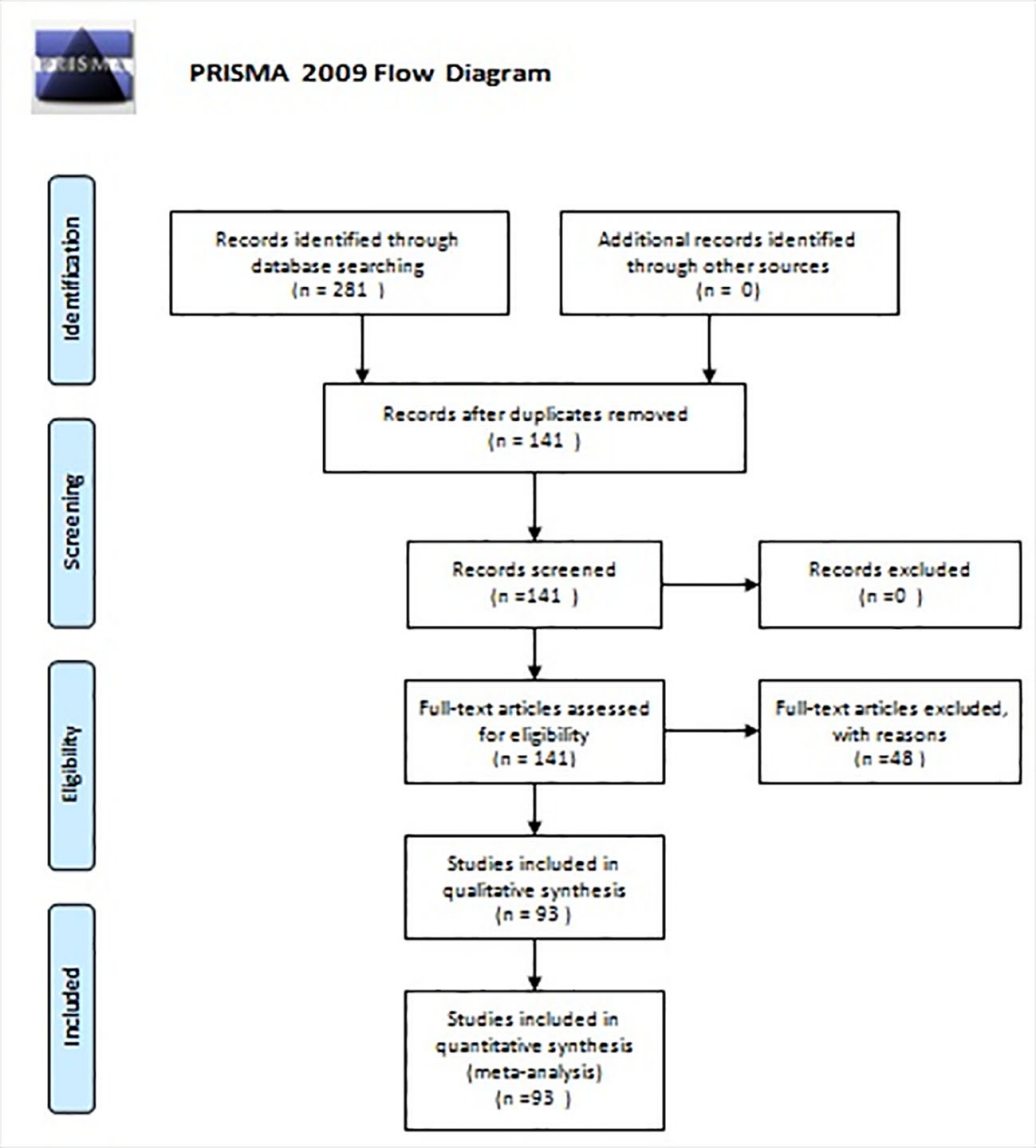
Flow diagram of the systematic review.

A bibliographic research was performed using Medline(PubMed), Scopus, Embase, and Google Scholar. The search terms employed were “parathyroid adenoma” or “primary hyperparathyroidism” and “pregnancy” or “postpartum”. Since 1953, when the first description of PA in pregnancy was made, 281 articles were published. In the latest 20 years (2000–2021), 141 articles were found. The primary objective of this study was to assess the treatment options in parathyroid adenoma during pregnancy regarding maternal and fetal complications. As secondary outcome, symptomatology and diagnostic options were considered. Inclusion criteria were all reports of cases of parathyroid adenoma in pregnancy and their treatment. Exclusion criteria were articles published before 2000 (due to missing data and lack of modern imaging), cases with hyperplasia or secondary hyperparathyroidism or parathyroid carcinoma, and reviews of the literature. In total, 93 articles were included in our review. **Figure 1** shows the flowchart of this review (**Figure 2**).

In order to express results, descriptive statistics were used appropriately. Means, medians, and standard deviations were used for continuous variables (4). The normal distribution of quantitative data were checked using the Kolmogorov–Smirnov test. Non-parametric tests (Mann–Whitney U test) were applied for the non-normal distribution data. T-test was used for normally distributed data. Chi-square tests have been applied for the data correlated. The ROC curve has been calculated for the detection of a cutoff point level. Excel 2007 and SPSS 22.0 were employed to statistically analyze the data.

## Results

Characteristics of parathyroid adenoma were determined concerning age, gestational week at diagnosis, ionized calcium levels, genetic testing result, symptomatology, radiological method of localization, treatment method, gestational week at operation, and maternal/fetal complications. In the literature searched, 211 cases of parathyroid adenoma in pregnancy were recorded.

**Figure 3:**
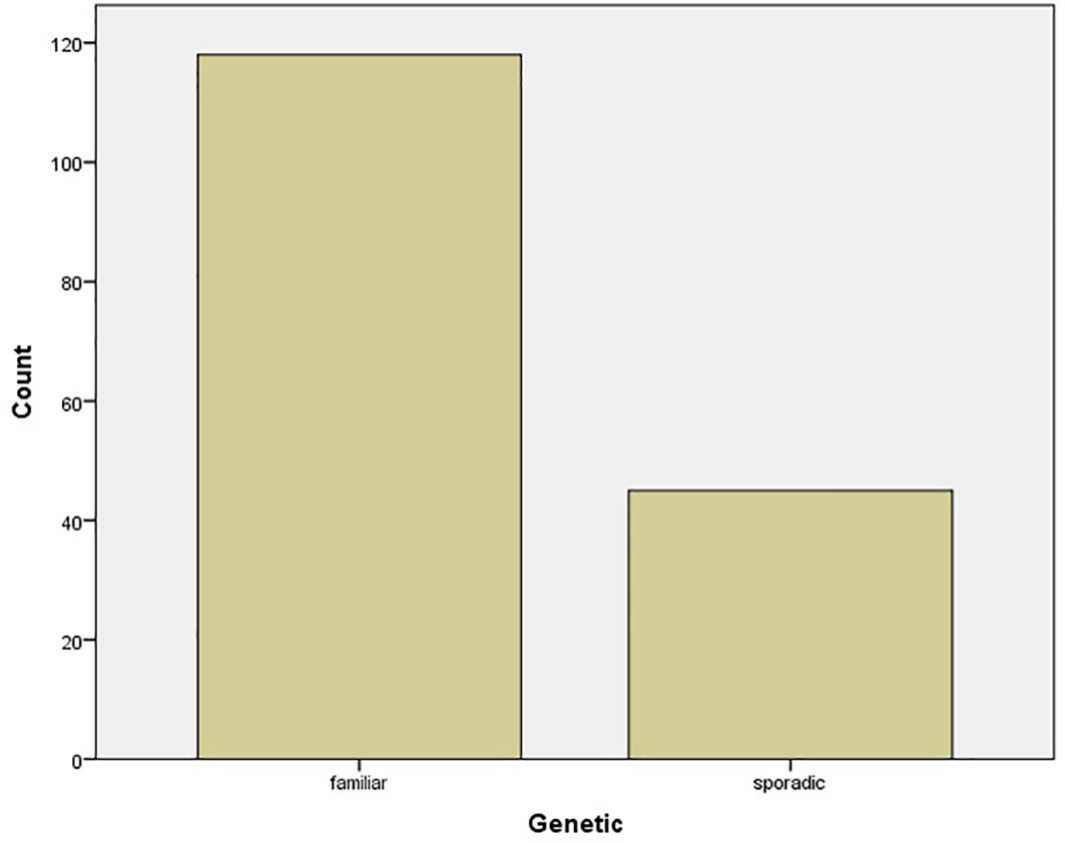
Bar chart of genetic testing results.

The mean age of pregnant women was 30.85 ± 5.57 years, ranging from 18 to 43 years. The median gestational week at diagnosis was 21 ± 9.61 weeks. The mean level of ionized calcium was 2.69 mmol/l [SD = 0.75 (2.55–2.84 95% CI)]. The genetic testing results showed that 6/51 (11.8%) of cases were familiar (all cases were MEN1), while 45/51 (88.2%) were sporadic (**Figure 3**).

**Figure 4:**
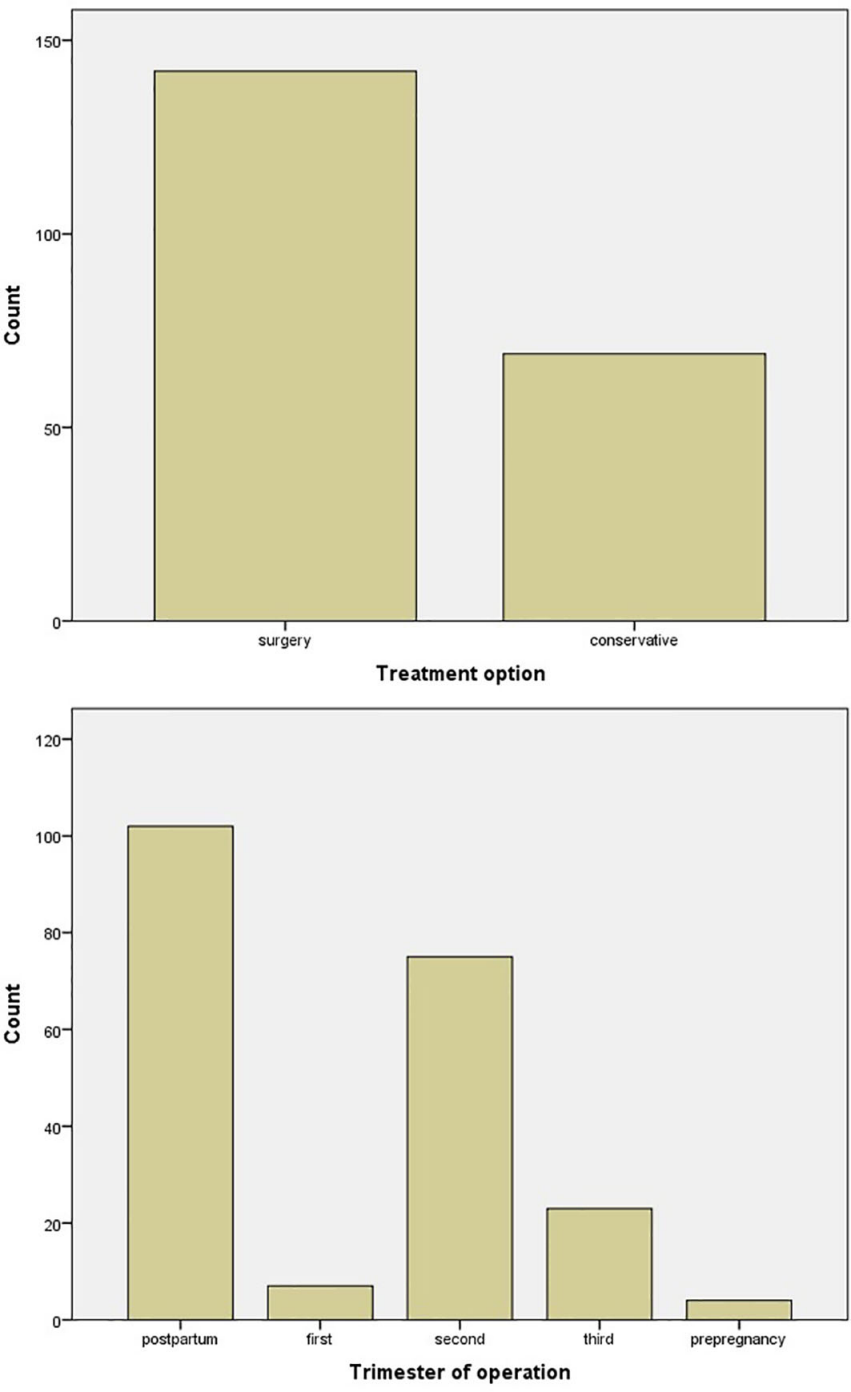
Bar chart of treatment options and trimester of operation.

**Figure 5:**
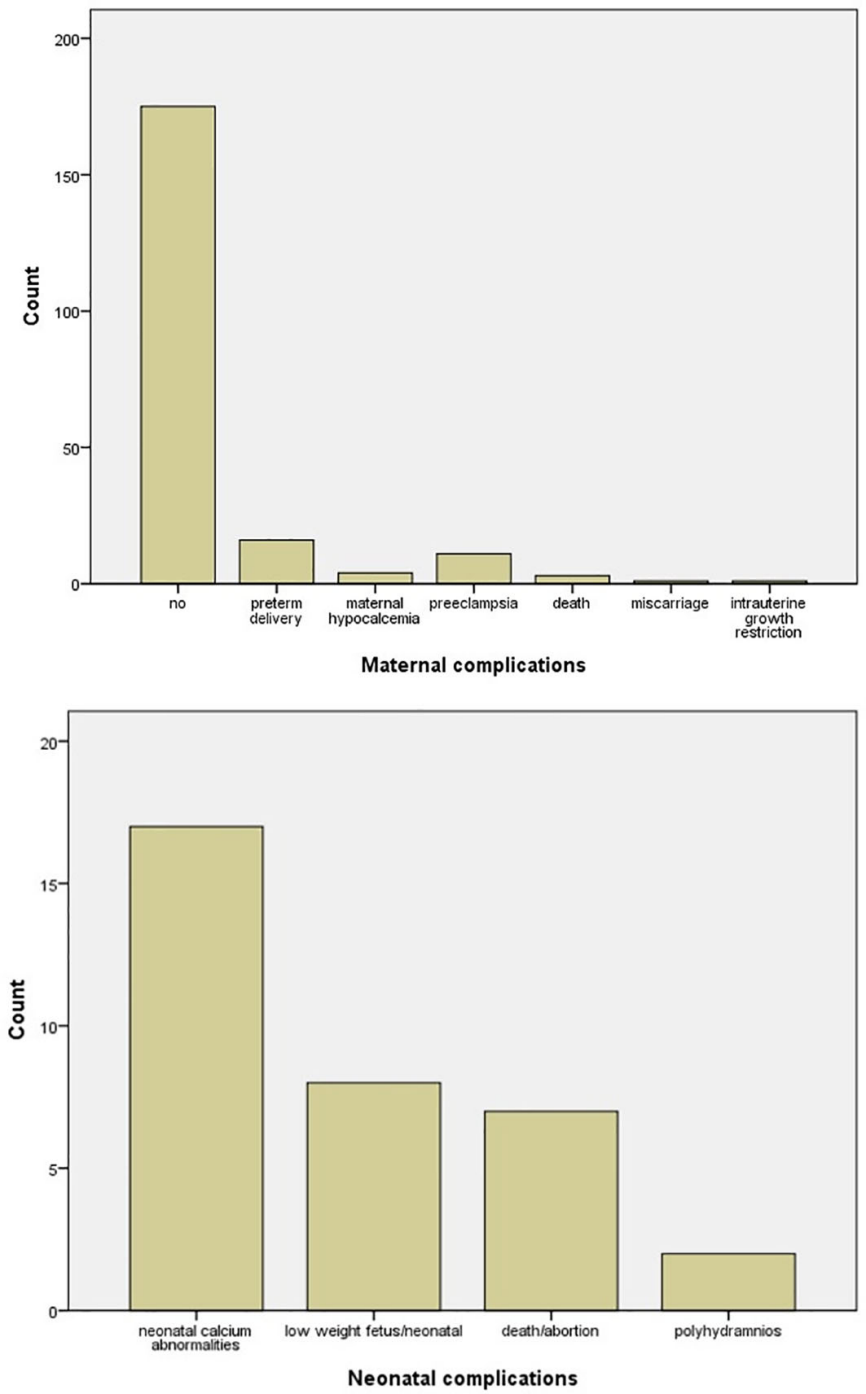
Bar chart of maternal and neonatal complications.

Surgery was the favorite treatment option with 142/211 (67.3%) in comparison with conservative treatment with 69/211 (32.7%), with either hydration, vitamin D supplementation, and steroid and furosemide administration or medical treatment with cinacalcet and calcitonin. In six cases, cinacalcet was administered, but surgery was afterward applied (**Figure 4**). The main gestational trimester of operation was the second one (35.5%), while in the first one 3.3% of pregnant patients were operated and in the third trimester 10.9%. A high percentage of operation was observed in the postpartum (48.3%) (**Figure 4**). Maternal and neonatal complications were observed in either conservative or surgical treatment group. No maternal complications were described in 82.6% of cases, while preterm delivery (7.6%) and preeclampsia (5.2%) were the most common complications (**Figure 5**). In the neonatal group, calcium abnormalities (in fact hypocalcemia) were most usually observed as a complication (50%), followed by low-weight fetus or newborn (23.6%) (**Figure 5**).

**Figure 6:**
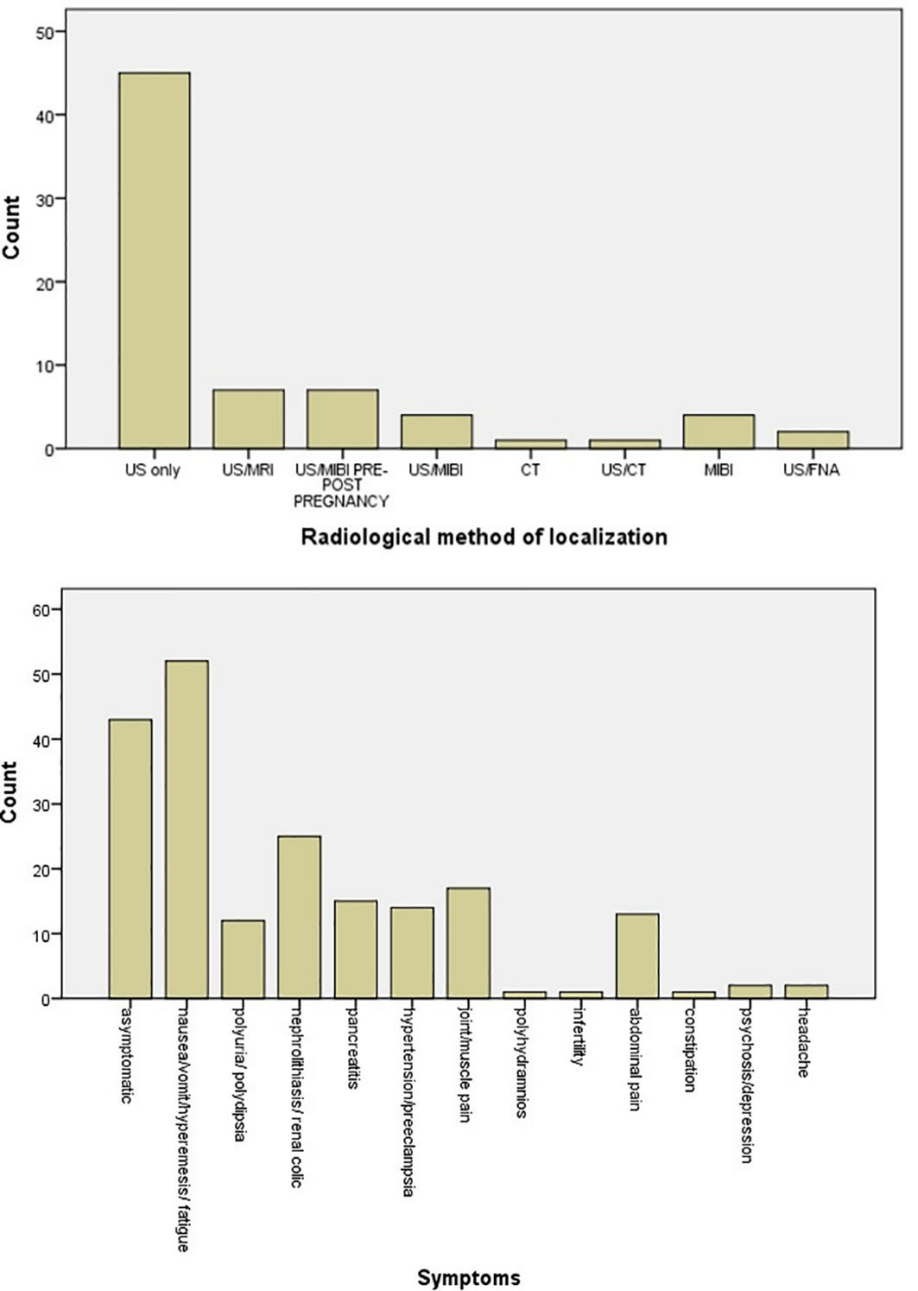
Bar chart of symptomatology of parathyroid adenoma in pregnancy and radiological methods applied for the localization of parathyroid adenoma.

The most common symptoms are nausea/vomit/hyperemesis/fatigue (26.3%) and symptomatology from urinary track (nephrolithiasis) (12.6%), while 21.7% of patients were asymptomatic. Joint/muscle pain (8.6%), pancreatitis (7.6%), hypertension/preeclampsia (7.1%), abdominal pain (6.6%), and polyuria/polydipsia (6.1%) follow in frequency (**Figure 6**). There was no statistical significant difference in calcium levels between symptomatic and asymptomatic diseases (p = 0.24).

The main radiological method applied for localization of the parathyroid adenoma was ultrasound (63.4%), while combination of ultrasound and MRI was used in 9.9%. Ultrasound and scintigraphy pre- or post-pregnancy were applied also in 9.9%. Scintigraphy alone during pregnancy, following the safety protocols, was used in 5.6% of cases (**Figure 6**).

The ionized calcium levels seemed to have a statistical significant correlation with the treatment method (Z = -2.593, p = 0.01). The cutoff point of the ionized calcium level suggestive of surgery was found 2.86 mmol/l by ROC curve analysis (AUC = 0.61, 95% CI [0.52, 0.69]). The complications were not correlated with the mother’s age (p = 0.91). The complication risk was associated with treatment option (p < 0.001), and yet by conservative treatment, the risk is 2.85 (95% CI [1.66–4.89]), making it more possible that complications will arise. A logistic regression was applied to analyze all risk factors for complications, but no significant predictor for complications was found by this model (age: p = 0.938, gestation week = 0.931, serum calcium levels: p=0.194, treatment option: p = 0.113, symptomatology: p = 0.567).

## Discussion

### Prevalence

The incidence of primary hyperparathyroidism in the general population is estimated about 0.15%–0.4%. Nowadays, the incidence approaches 1% due to the increase in serum calcium measurements routinely (5). This prevalence in pregnancy is calculated <1% in West countries, a low incidence rate, as primary hyperparathyroidism remains often undiagnosed in pregnant women. The etiology is that serum calcium measurement is not included in the routine gestational examination, while the normal changes in homeostasis of calcium metabolism in pregnancy may cover parathyroid adenoma diagnosis. Besides, the vast majority of pregnant women are asymptomatic or the symptoms overlap those of pregnancy (fatigue, nausea, vomiting) (5). In an Indian large cohort study, the prevalence of primary hyperparathyroidism in pregnancy was calculated relatively high, 2.1%, a fact that could be explained by the younger age of primary hyperparathyroidism appearance in East countries (6).

### Physiological maternal and fetal changes in calcium metabolism

The serum calcium and phosphate homeostasis as long as PTH and 1,25-dihydroxycholecalciferol regulation change during pregnancy. Maternal hemodilution due to plasma volume increases and hypoalbuminemia causes lower total serum calcium (6). On the contrary, the levels of ionized serum calcium remain unchanged and its measurement is a reliable mark of calcium in gestation. Furthermore, increased renal calcium excretion caused by increased glomerular infiltration results in maternal hypercalciuria. As a consequence, the risk for renal calculi in gestation is higher. The fetal requirement for calcium is 20–30 g for normal development and bone mineralization, passing through the placenta. The increased maternal absorption of calcium starts early at 12 weeks, and the pregnant remains in positive calcium balance until the third trimester, when there is a peak excretion of calcium. Calcium homeostasis is not regulated by maternal PTH, but by PTH-related protein, produced by the placenta, fetal tissues, and maternal breast. The PTH level in the first trimester is decreased to low-normal values and to mid-normal values in the second and third trimesters, whereas the PTH level does not mediate 1a-hydroxylase secretion, also regulated by PTH-related protein. 1,25-Dihydroxycholecalciferol (calcitriol) production, mediated by 1a-hydroxylase, is two- to three-fold elevated in pregnancy, regulated by PTHrp, estrogen, estradiol, prolactin, and lactogen. 1,25-Dihydroxycholecalciferol mediates the maternal intestinal reabsorption of calcium that is also increased in gestation. PTH, PTHrp, calcitriol, and calcitonin do not pass the placenta, while the fetus is dependent on maternal 25-hydroxyvitamin D, passing through the placenta. Fetal ionized calcium and phosphate are higher than maternal, essential for right skeletal development, and neonatal hypocalcemia is rare (5, 7).

### Clinical manifestations

The abovementioned physiological maternal changes in calcium metabolism can mask primary hyperparathyroidism diagnosis (6, 8). Nephrolithiasis and osteopenia are expected in gestation (5, 7). The main manifestations of primary hyperparathyroidism can mimic pregnancy symptoms. Mild primary hyperparathyroidism is usually asymptomatic, whereas non-specific symptoms such as fatigue, malaise, nausea, vomiting, dizziness, headache, constipation, polydipsia, polyuria, and bone and muscle aches are not indicative of primary hyperparathyroidism (5). Severe manifestations have been reported as complications of primary hyperparathyroidism. Acute or recurrent pancreatitis caused by hypercalcemia has been reported in high frequency in pregnant women (9–14). Pancreatitis should be excluded in cases of gestational hyperemesis (7, 15). Hyperemesis gravidarum due to primary hyperparathyroidism is reported in 21%–37% of cases (5). Gastrointestinal track symptoms seem to have a lead role in primary hyperparathyroidism in pregnancy (87.5% rate of appearance) in contrast to non-pregnant primary hyperparathyroidism cases (31.82%) (16). Urinary track symptoms (50%) and joint pain (50%) follow in frequency (16). Hypertensive crisis leading to preeclampsia is another severe complication of primary hyperparathyroidism during pregnancy. In a vast majority of women, preeclampsia was the main clinical manifestation of primary hyperparathyroidism (12, 17–20). In an Indian cohort study, the rates of pancreatitis and preeclampsia appearance were 50% and 88%, respectively (6). A case of uremic encephalopathy related to hypercalcemic crisis and a case of intracerebral hemorrhage were mentioned in the literature (20, 21). In cases of unexplained polyhydramnios, primary hyperparathyroidism should be excluded (5, 22). Miscarriage rates were three- to five-fold elevated in cases of undiagnosed primary hyperparathyroidism, and a correlation between higher levels of calcium >11.4 mg/dl and early pregnancy loss has been described (2, 22). Prior miscarriage has been reported at 62.5% according to Pal et al. (6). The exact relation between levels of serum calcium and the severity of primary hyperparathyroidism has not been determined yet. Some studies suggest that higher levels of calcium lead to more severe symptoms, whereas others support the fact that mid hypercalcemia cannot exclude severe complications (22, 23). On the other hand, a large retrospective study concluded that mild elevation of calcium is not associated with obstetrical complications (3). In our case report, the patient has second-trimester hyperemesis without previous symptoms, which made her gynecologist investigate the case, and hypercalcemia was the main clue to the diagnosis of parathyroid adenoma.

### Diagnosis

Normal changes in calcium metabolism also affect the diagnosis of primary hyperparathyroidism in pregnancy. The diagnosis of primary hyperparathyroidism in the general population is based on PTH and serum calcium elevated values and vitamin D and phosphorus level decrease. In pregnancy, serum total calcium is decreased, a fact that could mask primary hyperparathyroidism (7). The percentage of undiagnosed cases remains high—80% (7). The diagnosis during gestation is established by elevated serum ionized or albumin-corrected calcium levels with debatable PTH levels (5). The suspicion for primary hyperparathyroidism should always be made in cases of increased ionized calcium levels combined with symptomatology of vomiting, nausea, polyhydramnios, nephrolithiasis, and uncontrolled hypertension (22). It is crucial that familiar hypocalciuric hypercalcemia syndrome (FHH), which mimic primary hyperparathyroidism, is excluded (5). Familial hypocalciuric hypercalcemia is a rare autosomal dominant condition that causes mild hypercalcemia, hypocalciuria, hypermagnesemia, and hypophosphatemia. It occurs as a result of mutations in the calcium-sensing receptor gene (CASR) that lead to decreased receptor activity (24). Genetic testing is essential in cases of primary hyperparathyroidism in women <40 years old, so that multiple endocrine neoplasia (MEN) syndromes are excluded (5). Diagnostic algorithms have been proposed in the literature. Early diagnosis is vital, so when elevated calcium levels are detected, PTH levels should be measured. In case of PTH low, vitamin D oversupplementation, cancer, lymphoma/myeloma, prolonged immobilization, adrenal insufficiency, sarcoidosis, and hyperthyroidism should be evaluated. In case of PTH normal or high and urine calcium low, FHH should be excluded. In case of PTH normal or high and urine calcium high, primary hyperparathyroidism and multiple endocrine neoplasia are prominent diagnoses (25).

In the international literature, cases of coexistence of parathyroid adenoma and thyroid adenoma or carcinoma have been reported, suggesting that a preoperative detailed patient workup should be made, aiming the best preoperative preparation (26–28).

The localization method that should be applied in primary hyperparathyroidism during pregnancy is a debatable subject in literature. Ultrasonography remains the gold standard for the diagnosis and localization of parathyroid adenoma (25). Its sensitivity is 69% and specificity 74% in experienced hands, as it is a fully objective examination, while these percentages approach 100% when ultrasound is combined with FNA-guided PTH measurement of suspected nodule (5). FNA biopsy has been mentioned as a valuable technology tested in some cases of primary hyperparathyroidism during pregnancy (29, 30). Magnetic resonance imaging (MRI) is an acceptable alternative when ultrasound cannot localize the adenoma. MRI lacks sensitivity in small parathyroid adenoma, and in high-volume endocrine surgeons’ teams, it is not required (5, 31). Computed tomography and sestamibi should be avoided in gestation due to radiation exposure of fetus (25). These examinations are proposed in cases of negative bilateral neck exploration (5, 32). ^99m^Tc-MIBI scintigraphy has been suggested as a solution in these cases, but it is crucial that a reduction of 50% of radiation to 10 mCi is made so that the fetus is exposed to less than 5 mGy and the appropriate radiopharmaceutical is selected so that the best balance between the identification of parathyroid adenoma and the minimal fetal risk is achieved (33, 34).

A diagnostic and therapeutic algorithm for primary hyperparathyroidism during pregnancy cases has been suggested by Walker et al. In cases of suspicion of primary hyperparathyroidism, a multidisciplinary team should be approached and advised. Necessary biochemical examination (calcium, phosphorus, PTH, vitamin D) should be done, and in case of uncertainty, genetic testing follows. For the identification of parathyroid adenoma, ultrasound examination should be applied, and if the result is negative, bilateral neck exploration should be done. In case of ultrasound localization of parathyroid adenoma, minimally invasive parathyroidectomy is proposed. If neck exploration is negative, MRI, scintigraphy, intraoperative ultrasound, and 4D-CT are additional radiological examinations that could be helpful. A postoperative follow up for at least 6 months is proposed (35).

## Treatment

The definite treatment of primary hyperparathyroidism in the general population is surgical excision of the adenoma. In cases of primary hyperparathyroidism during pregnancy, there are no guidelines for the management of primary hyperparathyroidism, and it is controversial regarding the concerns for both mother and fetus arising from general anesthesia and surgery (5). Some treatment option criteria have been set as guidance for decision. The management of primary hyperparathyroidism during pregnancy should be based on gestational age, severity of hypercalcemia, and risk–benefit balance (7). A conservative treatment, including low-calcium diet, vitamin D supplementation, and hydration, should be chosen for mild to moderate hypercalcemia (ionized serum calcium < 2.75 mmol/l), whereas surgery is the treatment of choice in cases of symptomatic hypercalcemia. The most appropriate trimester for surgery is the second one, as in the first trimester fetal organogenesis takes part and in the third trimester the risk for preterm labor increases (5, 7, 31, 36). If the surgical option is declined, medical treatment with cinacalcet, bisphosphonates, and calcitonin could be proposed. Cinacalcet is a calcimimetic calcium-censoring receptor activator, decreasing PTH secretion by parathyroid glands. Cinacalcet is a category C drug in pregnancy, so its administration should be avoided (5, 34). Although it has been applied in restricted cases without side effects for both mother and fetus, further studies should be provided for its safety during pregnancy (10, 37, 38). Bisphosphonates are also a category C drug and are contraindicated in pregnancy due to the induction of fetal bone malformation (5). Calcitonin is a category B drug in pregnancy and seems to be ineffective and develops tachyphylaxis (5). As an alternative to medical treatment, a method with ethanol ablation of parathyroid adenoma by ultrasound guidance is described with acceptable results (6, 10).

The second trimester is considered the safest for surgery in gestation (7, 25, 39). However, there are studies that concluded the acceptance of surgery in the third trimester if risk–benefit balance is over benefit (22, 25, 36, 40–42). Some cases of parathyroidectomy without maternal or fetal complications in the first trimester have been also published (39, 41, 43–46).

As far as the operational strategy is concerned, in cases of a good preoperative ultrasound examination with high accuracy at parathyroid adenoma identification, minimally invasive parathyroidectomy is the treatment of choice (8, 23, 35, 47, 48). Minimally invasive parathyroidectomy could also be achieved by intraoperative ultrasound guidance (49). Minimally invasive surgery with cervical plexus block without recurrence and fetal complications has been described as an alternative to general anesthesia (23). In cases of negative preoperative radiological localization, bilateral neck exploration is mandatory (35). Regarding embryological features of parathyroid glands, their location may vary. Many cases of ectopic parathyroid adenoma have been reported, making the decision for surgery in pregnant women more difficult. In the majority of cases, adenoma was not identified preoperatively, the neck exploration was negative, and additional imaging methods were applied for their localization (50–53).

Parathyroidectomy is considered the treatment of choice regardless of pregnant patients’ symptomatology, as surgery lowers the risks of maternal and fetal complications in comparison to conservative treatment (16, 40). The possibility of preeclampsia is higher in conservative treatment (36). On the other hand, many cases of preeclampsia weeks after parathyroidectomy have been referred (8, 26, 54). A large retrospective study by Hirsch et al. concluded that there is no statistical significance of pregnancy-related complications between parathyroidectomy and conservative treatment (3).

The maternal complications due to untreated hypercalcemia have been analyzed above as clinical manifestations of primary hyperparathyroidism. The fetal complications include spontaneous abortion (15%), intrauterine fetal demise (7%), neonatal death (11%–16%), neonatal hypocalcemia-tetany (22%–50%), premature birth, intrauterine growth restriction, and low birth weight (5, 55).

A multidisciplinary team should manage primary hyperparathyroidism in pregnancy, and diagnosis and treatment algorithms should be established. If parathyroid adenoma is diagnosed before fertile and there is a high chance of pregnancy, parathyroidectomy is mandatory. During gestation, parathyroidectomy is suggested in the second trimester even in mild hypercalcemia. If an elevated serum ionized calcium (>2.43 mmol/l) is detected in early pregnancy or pre-pregnancy, PTH levels should be measured. In the postoperative course, serum calcium and PTH should be measured every 2 weeks so that hypocalcemia is avoided (39). In our case, conservative treatment with vitamin D supplementation was not a choice due to hypercalciuria and surgery was mandatory. The second trimester of pregnancy is the appropriate period for operation, as possible maternal and fetal anesthetic and operative risks are lesser.

## Conclusion

Primary hyperparathyroidism in pregnancy is a relatively rare condition that remains often undiagnosed. The similarity of primary hyperparathyroidism symptoms to those of pregnancy as long as of the normal changes taking place in pregnancy mask primary hyperparathyroidism diagnosis. Untreated hypercalcemia can lead to severe maternal and fetal/neonatal complications. Mild to moderate hypercalcemia can be treated conservatively, but severe or symptomatic hypercalcemia should be treated surgically. The benefits of the operation outweigh its risks, especially when it is undertaken in the second trimester of pregnancy. Further studies should be done so that medical drugs applied for hypercalcemia are evaluated for effectiveness and safety during pregnancy. A delay-onset or persistent hyperemesis is an important hint for hypercalcemia during pregnancy. Parathyroidectomy during pregnancy requires a highly specialized surgical team, and pre- and postoperative management of these patients is a multidisciplinary task.

## Data availability statement

The original contributions presented in the study are included in the article/**Supplementary Material**. Further inquiries can be directed to the corresponding author.

## Author contributions

(I) Conception and design: PI, PT, and CA. (II) Administrative support: KG, IA. (III) Provision of study materials or patients: PI, KT, MM. (IV) Collection and assembly of data: PA, CA. (V) Data analysis and interpretation: CA. (VI) Manuscript writing: all authors. (VII) Final approval of manuscript: all authors.

## Conflict of interest

The authors declare that the research was conducted in the absence of any commercial or financial relationships that could be construed as a potential conflict of interest.

## Publisher’s note

All claims expressed in this article are solely those of the authors and do not necessarily represent those of their affiliated organizations, or those of the publisher, the editors and the reviewers. Any product that may be evaluated in this article, or claim that may be made by its manufacturer, is not guaranteed or endorsed by the publisher.

## Supplementary material

Supplementary Table 1 Characteristics of cases included in the study. F, familiar; S, sporadic; US, ultrasound; MIBI, ^99m^TC-scintigraphy; CT, computed tomography; MRI, magnetic resonance imaging; C, conservative; S*, surgery.

## Abbreviations

PTH, parathormone; PA, parathyroid adenoma; PHPT, primary hyperparathyroidism; MEN, multiple endocrine neoplasia; FHH, familiar hypocalciuric hypercalcemia.
