# Supplementary material for: Parathyroid adenoma in pregnancy: A case report and systematic review of the literature
Source: Front Endocrinol (Lausanne). 2022 Oct 17;13:975954. doi: 10.3389/fendo.2022.975954 (PMC9618884; doi:10.3389/fendo.2022.975954)
Supplement: Supplementary file 1 [file Table_2.doc]

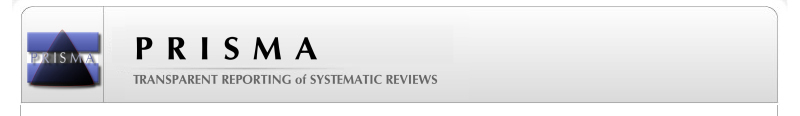
**PRISMA 2009 Flow Diagram**

**Screening**

**Included**

**Eligibility**

**Identification**

Records identified through database searching
(n = 281 )

Additional records identified through other sources
(n = 0)

Records after duplicates removed
(n = 141 )

Records screened
(n =141 )

Records excluded
(n =0 )

Full-text articles assessed for eligibility
(n = 141)

Full-text articles excluded, with reasons
(n =48 )

Studies included in qualitative synthesis
(n = 93 )

Studies included in quantitative synthesis (meta-analysis)
(n =93 )
