# Supplementary material for: Parathyroid adenoma in pregnancy: A case report and systematic review of the literature
Source: Front Endocrinol (Lausanne). 2022 Oct 17;13:975954. doi: 10.3389/fendo.2022.975954 (PMC9618884; doi:10.3389/fendo.2022.975954)
Supplement: Supplementary Table 1 — Characteristics of cases included in the study. F, familiar; S, sporadic; US, ultrasound; MIBI, 99mTC-scintigraphy; CT, computed tomography; MRI, magnetic resonance imaging; C, conservative; S*, surgery. [file Table_1.docx]

| **Author** | **No of cases** | **Age** | **Gestation week** | **Calcium** | **F/S** | **Symptoms** | **Past sym** | **Localization** | **C/S*** | **Oper week** | **Histology** | **Outcome** |
| --- | --- | --- | --- | --- | --- | --- | --- | --- | --- | --- | --- | --- |
| McCarthy et al (56) | 1 | 20 | 5 | 2,74 | F | nausea | no | US | S | 18 | Hyperplasia | No |
|  | 2 | 40 | 7 | 3,08 | S | asympt | no | US/ MIBI/CT PRE | S | 16 | Adenoma | preterm delivery |
|  | 3 | 34 | 16 | 2,79 | S | asympt | no | US | S | 27 | Adenoma | no |
| Ali et al(57) | 1 | 34 | 7 |  | F | infertility | no | US/MIBI | S | 24 | Hyperplasia | mat hypocalc/ fetal no |
| Malekar-Raikar et al(8) | 1 | 29 | 18 | 3,47 | S | nausea | no | US | S | 18 | Adenoma | preeclampsia |
| Herrera-Martinez(58) | 1 | 31 | 22 | 2,79 | S | asympt | no | US | S | 24 | Adenoma | no |
|  | 2 | 31 | 11 | 3,17 | S | asympt | nephrolit | US | S | 21 | Adenoma | No |
| Rchachi et al(59) | 1 | 26 | 33 | 3,12 |  | polyur/dips | no | us | S | 33 | Hyperplasia | hypocalc/preterm |
|  | 2 | 42 | 32 | 2,99 |  | nephrolit | no | us | S | 32 | Adenoma | hypocalc |
|  | 3 | 32 | 23 | 2,74 |  | nephrolit | no | us/MRI | C |  |  |  |
| Ning et al(60) | 1 | 37 | 32 | 1,49 |  | hypertension | HIV |  | C |  |  | preeclampsia/ neonatal hypocal |
| Perin et al(61) | 1 | 32 | 8 | 2,03 | S | nausea/weak | no | us/MRI | S | 15 | Adenoma | hypocalc |
| Guo et al(62) | 1 | 29 | 6 | 4,39 | F | nausea/weak | abortion | us/MIBI | S |  | Adenoma | no |
| Kokrdova et al(63) | 1 | 27 | 27 | 1,43 | S | nausea/weak | no | US | S | 31 | Adenoma | no |
| Sharma et al(64) | 1 | 25 | 30 | 2,77 | S | joint pain | no | US/MRI | C |  |  | preterm del/preecla/ neonatal low weight |
| Mokrysheva et al(65) | 1 | 28 | postpartum | 1,57 | S | joint pain | miscarriage/bone pain | CT | C | postpartum | Adenoma | hypocalc/ neonatal hypocalc |
| Zanardini et al(66) | 1 | 28 | 11 | 3,04 | S | nausea/weak | no | US/4D-CT | S | 14 | Adenoma | no |
| Muscat-Baron et al(67) | 1 | 26 | 34 | 3,2 |  | nephrolit/polyhydramnios | no | US | S | 34 | Adenoma | no |
| Truong et al(68) | 1 | 38 | 36 | 2,02 |  | flank pain | no | US | S | 36 | Adenoma | no |
|  | 2 | 35 | second trimester | 1,43 |  | nephrol | no | US/MIBI PRE | S | second trimester | double adenoma | no |
|  | 3 | 28 | 20 | 2,89 |  | nausea | PHPT first pregnancy/nephro/nausea | MIBI PRE | S | 20 | double adenoma | no |
| Nilsson et al(69) | 1 | 34 | 28 | 1,7 |  | asympt | nephrolit | US | S | 30 | Adenoma | no |
|  | 2 | 29 | 35 | 2,02 |  | premature delivery/nausea/hypertension | miscarriage/extrauterine pregnancy | US | C | postpartum | Adenoma | neonatal hypocalc |
| Jesudason et al(70) | 1 | 25 | 20 | 3,09 |  | renal colic | renal colic |  | S | 27 | Adenoma | no |
| Naru et al(71) | 1 | 23 | 22 | 3,17 |  | asympt | child seizures | US | S | 27 | Adenoma | no |
| Zhang et al(72) | 1 | 28 | 22 | 3,49 |  | nausea | no | US | S | 24 | triple adenoma | preterm del/low weight |
| Som et al(73) | 1 | 42 | 29 | 2,79 | S | hypertension | miscarriages | MIBI post | C | postpartum | Adenoma | neonatal death |
| Hession et al(74) | 1 | 33 | 28 | 3,3 |  | preeclampsia | miscarriages/ectopic pregnancy/renal calculi | US | C | postpartum | Adenoma | preterm delivery |
|  | 2 | 29 | 9 | 3 |  | abdominal pain/vomit | renal calculi/bone pain/lethargy | US | S | 19 | Adenoma | no |
| Mirza et al(44) | 1 | 31 | 6 | 3,52 |  | nausea/fatigue/polyur/myalgia/constipation | no | US | S | 7 | adenoma with atypia | no |
| Davis et al(54) | 1 | 35 | 13 | 3,6 | S | hyperemesis | constipation/ nephrolit | US | S |  | adenoma with atypia | preeclampsia/preterm del/neonatal low weight |
| Diaz-Soto et al(75) | 1 | 28 | 29 | 3,48 | S | nausea | pyelonephritis | US | S | 30 | adenoma | no |
|  | 2 | 40 |  | 3 | S | asympt | pre PHPT diagnosis | MIBI pre | C |  |  | no |
| Ince et al(76) | 1 | 34 |  | 2,74 | S | asympt | nephrolit | US/MIBI | S | 23 | adenoma | no |
| Godinez-Vidal et al(77) | 1 | 27 | 27 |  |  | pancreatitis | nephrolit |  | S |  | adenoma | no |
| Thomas et al(78) | 1 | 32 | 8 | 3,12 |  | hyperemesis/psychosis | no | US | S | 18 |  | no |
| Morrison et al(79) | 1 | 29 | 36 | 2,98 |  | asympt |  | US | C |  |  | no |
|  | 2 | 27 | 10 | 2,74 |  | asympt |  |  | C |  | adenoma |  |
|  | 3 | 20 | 24 | 3,1 |  | asympt |  | US | C |  |  | preterm delivery |
| Cuhaci et al(80) | 1 | 20 | 8 | 3,04 |  | abdominal pain |  | US | S | 9 | adenoma |  |
|  | 2 | 38 | 8 | 2,87 |  | asympt |  | US | S | second trimester | adenoma |  |
| Dogru et al(81) | 1 | 27 | 4 | 3,42 |  | nausea |  | US | S | second trimester | adenoma | no |
| Manjunatha et al(82) | 1 | 22 | 12 | 3,16 |  | hyperemesis |  | US | S | second trimester | adenoma | no |
| Gastelum et al(83) | 1 | 40 | 30 | 2,04 |  | abdominal pain |  | US | S | 30 | adenoma |  |
|  | 2 | 30 | 40 | 1,61 |  | preterm delivery |  | US | C | postpartum | adenoma |  |
| Sanderson et al(84) | 1 | 31 | 26 | 2,94 |  | flank pain |  | US | S | 26 | triple adenoma |  |
| Dokmetas et al(85) | 1 | 40 | 14 | 2,75 |  | asympt |  | US | S | 17 | adenoma |  |
| Bitar et al(86) | 1 | 33 | 15 | 3,27 |  | bone pain/nausea |  | US | S | 19 | adenoma | no |
| Baumann et al(26) | 1 | 23 | 31 | 3,07 |  | nausea/weak | no | US | S | 23 | adenoma | preeclampsia/preterm delivery |
| Li et al(27) | 1 | 27 | 16 | 3,48 | S | nausea | miscarriages | US/MRI | S | 18 | adenoma | no |
| Arnez et al(28) | 1 | 40 | 25 | 3,02 |  | polyur/dips | miscarriages | US | S | 28 | adenoma | no |
| Razavi et al(55) | 1 | 42 | postpartum | 2,84 |  | asympt |  | US | C | postpartum | adenoma | neonatal hypocal |
| Malheiro et al(33) | 1 | 31 | 16 | 2,95 |  | asympt | pre PHPT diagnosis | MIBI | S | 16 | adenoma | no |
| Pothiwala et al(29) | 1 | 30 | 8,5 | 3,37 |  | myalgias/bone pain/nausea | nephrolith | MRI/US/FNA | S | 11 | adenoma | no |
|  | 2 | 22 | 21 | 3,07 |  | asympt | intrauterine fetal death | MRI/US | S | 22 | adenoma | no |
| McMullen et al(32) | 1 |  | 8 | 2,7 |  | renal calculi |  |  | C | postpartum | adenoma | fetal death |
|  | 2 |  | 20 | 3,2 |  |  |  |  | C | postpartum | adenoma | preterm delivery |
|  | 3 |  | 34 | 2,7 |  |  |  |  | C | postpartum |  | preterm delivery |
|  | 4 |  | 38 | 3,1 |  | renal calculi |  | MIBI | C | postpartum |  | preterm delivery |
|  | 5 |  | 10 | 3,5 |  | renal calculi |  | MIBI | S | second trimester | adenoma |  |
|  | 6 |  | 21 | 2,8 |  |  |  |  | S | second trimester | adenoma |  |
|  | 7 |  | 23 | 3,2 |  | renal calculi |  | MIBI | S | second trimester | adenoma |  |
| Stringer et al(47) | 1 | 32 | 19 | 2,92 | S |  |  | US/MIBI | S |  | adenoma | no |
|  | 2 | 22 | 20 | 3,45 | F |  |  | US | S |  | adenoma | no |
|  | 3 | 30 | 22 | 2,78 | F |  |  | US | S |  | adenoma | no |
|  | 4 | 36 | 17 | 2,67 | S |  |  | US | S |  | adenoma | no |
|  | 5 | 31 | 23 | 3,09 | S |  |  | US | S |  | adenoma | no |
|  | 6 | 39 | 23 | 2,72 | S |  |  | US | S |  | adenoma | no |
|  | 7 | 37 | 32 | 3,06 | S |  |  | US | S |  | adenoma | no |
|  | 8 | 43 | 25 | 2,7 | S |  |  | US | S |  | adenoma | no |
| Dahan et al(9) | 1 | 22 | 32 | 2,89 |  | nausea/pancreatitis | no | US | C | postpartum | adenoma | preterm delivery |
| Bansal et al(10) | 1 | 31 | 24 | 3,09 |  | abdominal pain/vomit/ pancreatitis |  | US/MRI/FNA | C |  |  | no |
| Dias Leite et al(17) | 1 | 40 | 27 | 3,49 | S | nausea/ hypertension/pancreatitis |  | US | C |  |  | preterm delivery |
| Boorugu et al(11) | 1 | 27 | 26 | 3,37 | S | pancreatitis | no | US/MIBI post | C | postpartum | adenoma | neonatal hypocalc |
| Alharbi et al(18) | 1 | 28 | 27 | 2,97 |  | hypertension/preeclampsia | no | US | C |  |  | preterm delivery |
| Dale et al(12) | 1 | 31 | 32 | 1,58 |  | preeclampsia/ pancreatitis | no | US | C |  |  | preterm delivery |
| Alajmi et al(19) | 1 | 33 | 28 | 2,65 |  | hypertension | no | MIBI post | C | postpartum | adenoma | preterm delivery |
| Norman et al(2) | 1 | 19 |  | 2,87 |  |  | no |  | S | 17 | adenoma |  |
|  | 2 | 21 |  | 2,92 |  |  | miscarriage |  | S | 15 | adenoma |  |
|  | 3 | 24 |  | 2,82 |  |  | miscarriage |  | C |  |  |  |
|  | 4 | 25 |  | 2,75 |  |  | miscarriage |  | S | 14 | adenoma |  |
|  | 5 | 26 |  | 2,9 |  |  | miscarriage |  | C |  |  |  |
|  | 6 | 27 |  | 2,7 |  |  |  |  | C |  |  |  |
|  | 7 | 27 |  | 2,87 |  |  |  |  | S | 13 | adenoma |  |
|  | 8 | 27 |  | 2,8 |  |  | miscarriage |  | C |  |  |  |
|  | 9 | 28 |  | 2,97 |  |  | miscarriage |  | S | 15 | adenoma |  |
|  | 10 | 28 |  | 2,87 |  |  |  |  | S | 17 | adenoma |  |
|  | 11 | 28 |  | 2,62 |  |  |  |  | C |  |  |  |
|  | 12 | 29 |  | 2,82 |  |  | miscarriage |  | C |  |  |  |
|  | 13 | 29 |  | 2,8 |  |  | miscarriage |  | S | 23 | adenoma |  |
|  | 14 | 30 |  | 3,1 |  |  | miscarriage |  | C |  |  |  |
|  | 15 | 30 |  | 2,9 |  |  |  |  | S | 16 | adenoma |  |
|  | 16 | 31 |  | 2,85 |  |  | miscarriage |  | S | 14 | adenoma |  |
|  | 17 | 32 |  | 2,77 |  |  | miscarriage |  | C |  |  |  |
|  | 18 | 32 |  | 2,82 |  |  |  |  | S | 16 | adenoma |  |
|  | 19 | 33 |  | 2,8 |  |  |  |  | S | 14 | adenoma |  |
|  | 20 | 33 |  | 2,85 |  |  | miscarriage |  | C |  |  |  |
|  | 21 | 33 |  | 2,92 |  |  | miscarriage |  | C |  |  |  |
|  | 22 | 33 |  | 2,67 |  |  | miscarriage |  | C |  |  |  |
|  | 23 | 34 |  | 2,85 |  |  | miscarriage |  | C |  |  |  |
|  | 24 | 34 |  | 2,75 |  |  | miscarriage |  | C |  |  |  |
|  | 25 | 36 |  | 2,82 |  |  |  |  | S | 18 | adenoma |  |
|  | 26 | 36 |  | 2,97 |  |  | miscarriage |  | S | 16 | adenoma |  |
|  | 27 | 36 |  | 2,72 |  |  | miscarriage |  | C |  |  |  |
|  | 28 | 37 |  | 3,05 |  |  | miscarriage |  | S | 13 | adenoma |  |
|  | 29 | 37 |  | 3,25 |  |  | miscarriage |  | C |  |  |  |
|  | 30 | 38 |  | 3,07 |  |  | miscarriage |  | C |  |  |  |
|  | 31 | 38 |  | 2,97 |  |  | miscarriage |  | S | 17 | adenoma |  |
|  | 32 | 40 |  | 2,85 |  |  | miscarriage |  | C |  |  |  |
| Nash et al(21) | 1 | 30 | 32 | 3,2 |  | abdominal pain/vomit | miscarriage/nephrolith/ectopic pregnancy | US/MIBI post | C | postpartum | adenoma | preterm delivery |
| Krysiak et al(13) | 1 | 35 | 8 | 2,6 |  | pancreatitis | miscarriage | CT post | C | postpartum | adenoma | neonatal hypercalc |
| Tsai et al(15) | 1 | 31 | 15 | 2,99 |  | nausea/polyur/malaise/ pancreatitis | nephrolith | US | S | 16 | adenoma | no |
| Yang et al(14) | 1 | 24 | 37 | 3,11 | S | pancreatitis | no | CT/MIBI post | C | postpartum | adenoma | preterm delivery/ maternal death |
| Hong et al(20) | 1 | 32 | 37 | 1,59 |  | preeclampsia/ intracranial hemorrhage | no | US/CT post | C | postpartum | adenoma | preterm delivery |
| Petousis et al(87) | 1 | 28 | 26 | 2,99 | S | abdominal pain | no | US/MRI | S | 29 | adenoma | no |
| Dincer et al(50) | 1 | 29 | postpartum | 3,24 |  | muscle pain/pancreatitis |  | US/MRI/CT/MIBI post | C | postpartum | adenoma | no |
| Bendinelli et al(48) | 1 | 38 | 9 | 2,61 | S | polydip-ur/hyperemesis | no | US | S | 23 | adenoma | no |
| Tachamo et al(45) | 1 | 32 | 7 | 1,67 | S | lumbar pain | pituitary adenoma | US/FNA | S | first trimester | adenoma |  |
| Hui et al(41) | 1 | 35 | 28 | 3,08 |  | polur-dip | hypertension first pregnancy | US | S | 33 | adenoma | no |
|  | 2 | 29 | 10 | 3,18 |  | vomit |  | US | S | 11 | adenoma | no |
|  | 3 | 39 | 15 | 3,2 |  | asympt | PHPT pre diagnosis | US | S | 22 | adenoma | no |
| Sharma et al(43) | 1 | 31 | 6 | 3,52 |  | polyu-nausea-weak |  | US | S | 7 | adenoma with atypia | preeclampsia |
| Abusabeib et al(52) | 1 | 38 | 15 | 3,03 |  | joint pain | lumbar pain/lymphadenopathy | US/ CT/MIBI pre | S | 18 | adenoma |  |
| Horton et al(53) | 1 | 21 | 10 | 1,69 |  | vomit/polydip/headache |  | 4DCT | S | 14 | adenoma | preeclampsia |
| Osuna et al(46) | 1 | 22 | 8 | 3,87 |  | nausea/vomit/abdominal pain | hypertension/nephroloth | US | S | 8 | adenoma | no |
| Gonzalo-Garcia et al(38) | 1 | 30 | 30 | 3,67 |  | preeclampsia/intrauterine growth restriction | miscarriage | US/MRI-CT/MIBI post | C |  |  | preeclampsia/ preterm delivery/ neonatal hypocal |
| Saad et al(51) | 1 | 18 | 23 | 3,15 |  | weak/pain | nephrolit/polyur/vomit/weak | US/MIBI | S | 24 | adenoma | no |
| Haciyanli et al(49) | 1 | 25 | 19 | 2,94 |  | nausea/muscle pain | no | US | S |  | adenoma | no |
|  | 2 | 36 | 24 | 3,09 |  | asympt | no | US | S |  | adenoma | no |
| Vera et al(37) | 1 | 34 | 10 | 3,02 | S |  | nephrolith | US/MIBI/CT pre- US/FNA | C | postpartum | adenoma | neonatal hypocalc |
| Pal et al(6) | 1 | 26 | 28 | 2,87 |  | bone pain/renal colic |  | US | C | postpartum | adenoma | neonatal hypocalc |
|  | 2 | 38 | 20 | 3,02 |  | renal colic/weak | miscarriage/infertility | US | C | postpartum | adenoma | no |
|  | 3 | 36 | 16 | 2,59 |  | pancreatitis | miscarriage | US | C | postpartum | adenoma | no |
|  | 4 | 37 | 25 | 3,09 |  | bone pain/renal colic | miscarriage/PA excision | US | C | postpartum | double adenoma | no |
|  | 5 | 28 | 7 | 3,04 |  | cerebral venous thrombosis | nephrolith | US | C | postpartum | normal PGs | no |
|  | 6 | 32 | 28 | 2,99 |  | pancreatitis | nephrolith/ miscarriage | US | C | postpartum |  | no |
|  | 7 | 32 | 24 | 3,64 |  | pancreatitis | nephrolith/infertility | US | C | postpartum |  | no |
|  | 8 | 34 | postpartum | 2,82 |  | asympt | miscarriage | US | C | postpartum | adenoma | neonatal hypocalc |
| Song et al(16) | 1 | 29 | 5 | 1,73 |  | nausea |  |  | S | 18 | adenoma | no |
|  | 2 | 35 | postpartum | 1,69 |  | joint pain |  |  | C | postpartum | adenoma | neonatal hypocalc |
|  | 3 | 29 | postpartum | 1,36 |  | joint pain |  |  | C | postpartum | carcinoma | neonatal hypocalc |
|  | 4 | 28 | 14 | 1,7 |  | nausea |  |  | S | 15 | adenoma with atypia | no |
|  | 5 | 35 | 12 | 2,1 |  | nausea |  |  | S | 18 | adenoma | intrauterine death |
|  | 6 | 31 | 27 | 1,8 |  | nausea/ unconsiousness |  |  | S | 28 | adenoma | pancreatitis/ preeclampsia/HELLP |
|  | 7 | 35 | 8 | 1,34 |  | polydips |  |  | C |  |  |  |
|  | 8 | 27 | 12 | 1,46 |  | asympt |  |  | C |  |  | neonatal heart disease |
| DiMarco et al(31) | 1 | 37 |  | 3,04 | S | asympt |  | US/MIBI PRE | S | 19 |  |  |
|  | 2 | 35 |  | 3 | S | headache |  | US | S | 18 |  |  |
|  | 3 | 37 |  | 2,63 | S | asympt |  | US | S | 19 |  | preterm delivery |
|  | 4 | 40 |  | 2,78 | S | miscarriage |  | US/MIBI PRE | S | miscarriage |  | miscarriage |
|  | 5 | 40 |  | 2,74 | S | hypertension |  | US | S | 19 |  |  |
|  | 6 | 41 |  | 2,8 | S | asympt |  | US | S | second trimester |  |  |
|  | 7 | 30 |  | 2,56 | S | asympt |  | US/MIBI PRE | S | 24 |  |  |
|  | 8 | 32 |  | 3 | S | asympt |  |  | S | 28 |  |  |
|  | 9 | 36 |  | 2,66 | S | asympt |  | US | S | 19 |  |  |
|  | 10 | 33 |  | 3,3 | S | asympt |  | US | S | second trimester |  |  |
|  | 11 | 25 |  | 3,16 | F | MEN1 |  | US/MIBI PRE | S | 14 |  |  |
|  | 12 | 52 |  | 3 | S | asympt |  | US | S | 21 |  |  |
|  | 13 | 37 |  | 2,75 | S | renal calculi |  | US | S |  |  | intrauterine growth restriction |
|  | 14 | 29 |  | 3,35 | S | nausea |  | US | S | 12 |  |  |
|  | 15 | 32 |  | 2,85 | S | IVF workup |  | US/MIBI PRE | S | 24 |  |  |
|  | 16 | 34 |  | 2,88 | S | asympt |  | US | S | second trimester |  |  |
|  | 17 | 27 |  |  | S | asympt |  | US/MIBI PRE | S | second trimester |  |  |
| Walker et al(35) | 1 | 31 | prepregnancy | 2,88 |  | asympt | Li-fraumeni syndrome | US/MIBI PRE | S | second trimester |  |  |
|  | 2 | 24 | second trimester | 3,64 |  | nephrolit |  | US | S | second trimester |  |  |
|  | 3 | 29 | second trimester | 3,29 |  | nephrolit | HIV | US | S | second trimester |  |  |
|  | 4 | 28 | second trimester | 2,78 |  | asympt | Crohn's disease | US | S | second trimester |  |  |
|  | 5 | 29 | second trimester | 3,18 |  | abdominal pain |  | US | S | second trimester |  | hypercalcemia/neonatal hypercalcemia |
| Cassir et al(22) | 1 |  | 17 | 2,8 |  | polydips/weak/vomit | hypertension/ urinary track infections |  | S | 33 |  | no |
|  | 2 |  | 2 | 1,4 |  | renal calculi/weak/depression | nephrolith/ IBS |  | S | 26 |  | no |
|  | 3 |  | prepregnancy | 2,3 |  | renal calculi | prolactinoma/ MEN1 |  | S | prepregnancy |  | pregnancy termination |
|  | 4 |  | 10 | 3,1 |  | hyperemesis |  |  | S | 16 |  | low weight fetus |
|  | 5 |  | prepregnancy | 2,6 |  | renal calculi | hypothyroidism |  | S | prepregnancy |  | low weight fetus |
|  | 6 |  | prepregnancy | 2,7 |  | renal calculi/ muscle weak/fatigue | hypothyroidism/ osteopenia |  | S | prepregnancy |  | low weight fetus |
|  | 7 |  | prepregnancy | 2,5 |  | asympt | hypothyroidism/ radioactive iodine |  | S | prepregnancy |  | preeclampsia |
|  | 8 |  | 25 | 2,9 |  | fatigue/polydisp |  |  | S | 30 |  | no |
|  | 9 |  | 27 | 2,8 |  | asympt |  |  | S | 31 |  | no |
|  | 10 |  | 11 | 2,7 |  | renal calculi |  |  | S | 16 |  |  |
|  | 11 |  | 20 | 2,9 |  | hyperemesis | nephrolith |  | S | 22 |  | no |
|  | 12 |  | 34 | 2,9 |  | hyperemesis/ abdominal pain | bipolar disease |  | S | 35 |  | no |
|  | 13 |  | 12 | 3,1 |  | hypertension | depression |  | S | 13 |  | low weight fetus |
|  | 14 |  | 33 | 2,7 |  | asympt |  |  | C |  |  | polyhydramnios |
|  | 15 |  | 30 | 3,1 |  | polyur/poydips | hypertension |  | S | 34 |  | no |
|  | 16 |  | 25 | 2,9 |  | abdominal pain |  |  | C |  |  | no |
|  | 17 |  | 19 | 3,1 |  | abdominal pain/hyperemesis |  |  | S | 27 |  | hypertension |
|  | 18 |  | 31 | 3,6 |  | asympt | nephrolit |  | S | 32 |  | polyhydramnios |
|  | 19 |  | 15 | 3,7 |  | hyperemesis | depression |  | S | 16 |  | low weight fetus |
| Hu et al(23) | 1 | 37 | 7 | 1,34 |  | asympt |  |  | S |  | adenoma with atypia |  |
|  | 2 | 31 | 25 | 1,79 |  | asympt |  |  | S |  | adenoma with atypia |  |
|  | 3 | 28 | 11 | 1,75 |  | nausea |  |  | S |  | adenoma |  |
|  | 4 | 29 | 5 | 1,73 |  | nausea |  |  | S |  | adenoma |  |
|  | 5 | 32 | 27 | 2,29 |  | pancreatitis/nephrolith/hypertension |  |  | S |  | adenoma | abortion |
|  | 6 | 22 | 24 | 1,63 |  | headache/unconsiousness/ hypertension |  |  | S |  | adenoma | abortion |
|  | 7 | 35 | 18 | 3,07 |  | nausea/ nephrolit |  |  | S |  | adenoma | abortion |
|  | 8 | 34 | 6 | 3,15 |  | vomit/unconsiousness/ nephrolith |  |  | S |  | adenoma | abortion |
|  | 9 | 26 | postpartum | 1,39 |  | asympt |  |  | C | postpartum | hyperplasia | neonatal hypocalc |
|  | 10 | 28 | postpartum | 1,47 |  | asympt |  |  | C | postpartum | adenoma | neonatal hypocalc |
|  | 11 | 26 | postpartum | 1,48 |  | backache/vomit/ nephrolith/ hypertension |  |  | C | postpartum | adenoma | neonatal hypocalc |
|  | 12 | 29 | postpartum | 1,36 |  | asympt |  |  | C | postpartum | carcinoma | neonatal hypocalc |
| Jiao et al(39) | 1 | 37 | postpartum | 2,87 |  | asympt |  |  |  |  |  |  |
|  | 2 | 32 | prepregnancy | 2,95 |  | joint pain |  |  |  |  |  |  |
|  | 3 | 27 | second trimester | 3,28 |  | nausea/weak |  |  |  |  |  |  |
|  | 4 | 38 | second trimester | 4,21 |  | nausea |  |  |  |  |  |  |
|  | 5 | 34 | prepregnancy | 2,77 |  | nausea/weak |  |  |  |  |  |  |
|  | 6 | 28 | second trimester | 3 |  | nausea/weak |  |  |  |  |  |  |
|  | 7 | 25 | second trimester | 3,49 |  | nausea/weak |  |  |  |  |  |  |
|  | 8 | 30 | second trimester | 3,17 |  | joint pain |  |  |  |  |  |  |
|  | 9 | 32 | first trimester | 3,08 |  | nausea/weak |  |  |  |  |  |  |
| Rey et al(88) | 1 | 40 | 28 | 1,44 |  | bone pain/polyur | sickle cell disease | US | C | postpartum | adenoma | no |
|  | 2 | 30 | 36 | 1,68 |  | nephrolit | nephrolith | MIBI post | C | postpartum | adenoma | no |
|  | 3 | 26 | 14 | 2,05 |  | nausea/ joint pain | fibroma |  | C | postpartum | fibroma | pregnancy termination |

***Table 1.*** Characteristics of cases included in the study. Abbreviations: F;familiar, S;sporadic, US;ultrasound, MIBI;^99m^TC-scintigraphy, CT; computed tomography, MRI; magnetic resonance imaging, C; conservative, S*;surgery
